# Application of a Machine Learning Method for Prediction of Urban Neighborhood-Scale Air Pollution

**DOI:** 10.3390/ijerph20032412

**Published:** 2023-01-29

**Authors:** Ka-Ming Wai, Peter K. N. Yu

**Affiliations:** Department of Physics, City University of Hong Kong, Hong Kong SAR, China

**Keywords:** urban environment, air quality model, machine learning, ENVI-met model, smart city

## Abstract

Urban air pollution has aroused growing attention due to its associated adverse health effects. A model which could promptly predict urban air quality with considerable accuracy is, therefore, important and will benefit the development of smart cities. However, only a computational fluid dynamics (CFD) model could better resolve the dispersion behavior within an urban canyon layer. A machine learning (ML) model using the Artificial Neural Network (ANN) approach was formulated in the current study to investigate vehicle-derived airborne particulate (PM_10_) dispersion within a compact high-rise-built environment. Various measured meteorological parameters and PM_10_ concentrations were adopted as the model inputs to train the ANN model. A building-resolved CFD model under the same environmental settings was also set up to compare its model performance with the ANN model. Our results showed that the ANN model exhibited promising performance (r = 0.82, fractional bias = 0.002) when comparing the > 1000 h PM_10_ measurements. When comparing the diurnal hourly measured PM_10_ variations in a clear-sky day, both the ANN and CFD models performed well (r > 0.8). The good performance of the CFD model relied on the knowledge of the in situ diurnal traffic profile, the adoption of suitable mobile source emission factor(s) (e.g., from MOBILE 6 and COPERT4), and the use of urban thermal and dynamical variables to capture PM_10_ variations in both neutral and unstable atmospheric conditions. These requirements/constraints make it impractical for daily operation. On the contrary, the ML (ANN) model adopted here is free from these constraints and is fast (less than 0.1% computational time relative to the CFD model). These results demonstrate that the ANN model is a superior option for a smart city application.

## 1. Introduction

The epidemiologic evidence of particulate pollution-induced health effects is well documented [1,2]. A total economic loss of USD 2.4 billion per year was estimated from PM_10_-induced premature death and chronic respiratory diseases in the Pearl River Delta of southern China [3]. Road-side vehicular emissions are the main source of atmospheric particulates in the ambient urban air of cities that are not directly influenced by industrial emissions [4,5]. Hong Kong, a megacity in southern China, suffers from a similar air quality problem [6]. In view of this, a simulation model for urban air pollution, which can produce rapid and robust results, is of urgent need for practical use. It would benefit not only Hong Kong but also other megacities around the world. For instance, more than 50% of people live in cities in China [7]. Technological advances in urban air quality management in the context of simulations and monitoring are also essential for smart city development [8]. 

The plume dispersion in the urban canopy layer (UCL) is unique compared to that in the free atmosphere well above the UCL. The UCL is featured with building-induced flows, such as wake recirculation, channeling and branching in intersections. In addition, the heterogeneity in building heights could result in the asymmetries of the vertical plume structure and, in turn, a shift of the effective source height [9]. The Gaussian dispersion model offers a simplified representation of downwind concentration spread from the emission sources. Popular models of this type, which parameterize the urban effects, are the US EPA’s model AERMOD [10,11] and the UK’s ADMS-urban [12,13]. A CFD model is capable of better resolving building-influenced wind and turbulence mixing in the built environment, which governs the pollutant dispersion in the urban canopy layer, and is thus a more accurate method. However, both the computational resource and time for a CFD model are demanding [14,15,16,17]. In addition, atmospheric stratification, which governs the vertical motions of fluid particles, is one of the challenges in CFD simulations. Currently, many studies only focus on neutral flows because of their numerical simplification.

More recently, ML technique has been used in predicting regional-scale air pollution. A few studies reported better performance for ML models in regional-scale air quality prediction compared to conventional physiochemical numerical air quality models [18]. Various ML algorithms have been used in air pollution prediction, namely, ANN (Artificial Neural Network; [19]), LASSO regression (Least Absolute Shrinkage and Selection Operator regression; [20]), LSTM (Long Short-Term Memory; [21]), kNN (k-Nearest Neighbor; [22]), RF (Random Forest; [23]), and SVM (Support Vector Machines; [24]). Bozdag et al. [19] reported that ANN algorithm [among other algorithms (LASSO, SVR, RF, kNN)] produces the best results (r^2^ = 0.58; RMSE = 20.8, MAE = 14.4) when performing a spatial prediction of PM_10_ concentration in Turkey. Studies have shown that meteorological characteristics could play an important role in the prediction of air pollutants [21,25]. Ma and Zhang [26] commented that using some traditional algorithms, such as radical basis function, back propagation neural network and SVM model, requires too many inputs, but the prediction results are not in good agreement with the measurements. Nevertheless, an application of a ML model on neighborhood-scale air pollution dispersion within the UCL of a compact city is rarely found in the literature.

The study goal here is to investigate if a recently developed ML technique is feasible to build a fast and relatively accurate model to predict neighborhood-scale PM_10_ concentration levels in a compact-city environment. The performance of the ML model was compared with the PM_10_ measurements and then with a CFD model, which is known to provide more accurate results in predicting the PM_10_ levels in the UCL. Prior to the simulations, the ML model was formulated by a dataset of past PM_10_ monitoring data. The CFD model was set up with the environmental settings (e.g., building configurations) in the study area. 

## 2. Materials and Methods

**Figure 1:**
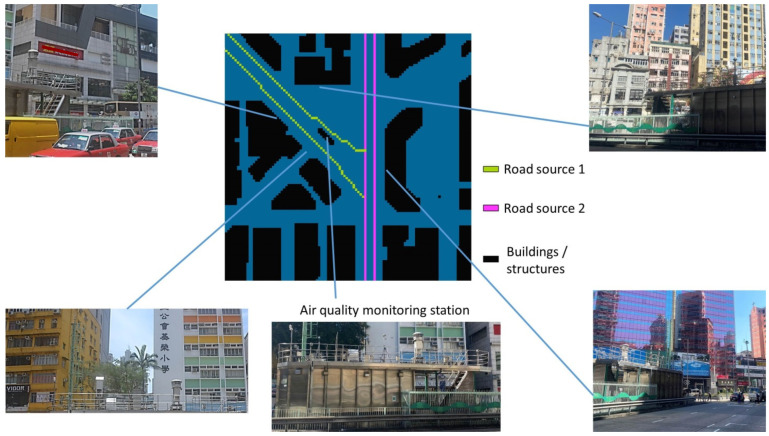
The site environment. The CFD model domain (center) and snapshots around the site are shown.

This study was conducted within a densely populated urban environment of Hong Kong (22.30° N, 114.17° E). The study site (Figure 1) is featured with a road-side air quality monitoring station operated by the Hong Kong Environmental Protection Department (EPD), two major roads, and sparse vegetation, and it is surrounded by buildings with different heights (5–26 stories). The subsequent section details the ANN and CFD models used here.

### 2.1. Artificial Neural Network (ANN) Model

**Figure 2:**
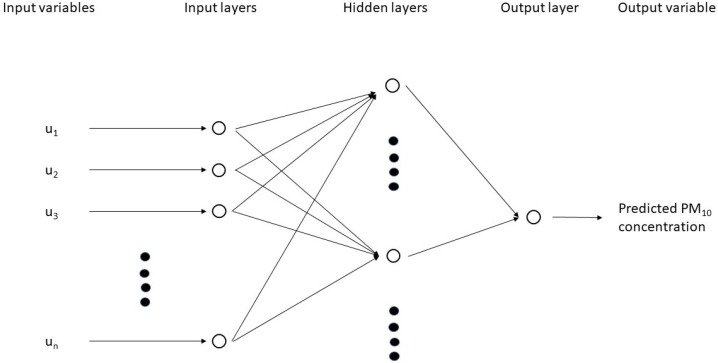
A schematic representation of the feed-forward neural network (FFNN).

The ANN (an ML algorithm) model was formulated to predict the neighborhood-scale PM_10_ dispersion within the UCL of the study site. The model mimics natural neurons in animal brains. The details of the model have been discussed elsewhere, e.g., [27]. Briefly, the ANN model consists of interconnected neurons at the input, hidden, and output layers (Figure 2). Input values are collected in the input layer and then sent to different neurons (or processing units), which constitute the hidden layer. Output variables are eventually obtained at the output layer after the data are processed. Each neuron in the hidden layer computes a weighted sum of the inputs. The weight is subjected to change during the ANN training in order to provide its best estimate to the output. The selection of a proper number of hidden layer is important for the model construction. Although adding more hidden layer might improve the model’s performance, it is noted that more complexity of the training process is imposed [28]. Therefore, one hidden layer was used here. The number of neurons in the hidden layer was determined by N_hidden_ = 2 N_input_ + 1, where N_hidden_ and N_input_ are the number of neurons in the hidden and input layers, respectively [29]. To avoid model instability, all input parameters were scaled from 0 to 1. The feed-forward neural network was used, which was successfully adopted in other pollution transport studies, e.g., [30]. It is called the feed-forward network since data flow within the network from one layer to the next one without any return path. A hyperbolic tangent sigmoid transfer function for the neurons in the hidden layer was adopted to reduce the computational time required during the training process. The efficient Levenberg–Marquardt algorithm for training was used, such that the model achieved a mean squared error (MSE) < 0.004. The model was constructed by the MATLAB software (The MathWorks, USA). Table 1 details the model settings.

### 2.2. Computational Fluid Dynamics (CFD) Model

The ENVI-met model (version 5.0) was used to simulate the PM_10_ dispersion in the UCL of the study area. It is a 3-dimensional, microscale, non-hydrostatic computational fluid dynamics (CFD) model and uses the RANS (Reynolds-Averaged Navier–Stokes) equations to simulate surface–plant–air interactions. The Boussinesq approximation was adopted for the thermal-forced vertical motion. The model description is detailed in Bruse and Fleer [31]. It has been used to study the atmospheric dispersion of air pollutants included in urban environments [32,33]. Particle sedimentation due to gravity and particle deposition to different surfaces by considering the aerodynamic and sub-layer surface resistances [34] were simulated. The simulation domain covered an area of 100 m × 100 m. A horizontal grid resolution was set as 2 m with 6 nesting grids at each border to avoid the edge effects. For vertical grids, the grid size varied from 20 cm in the first 1 m to a telescoping factor of 20% after a height of 1 m above ground.

The hourly wind speed, wind direction, and air temperature measured at the EPD’s air quality monitoring station (AQMS) were adopted as the model inputs [or the inflow boundary condition (BC)]. The hourly measured relative humidity was obtained from a nearby weather station. The wind speed at 10 m above ground, as required by the model, was derived by the following power-law equation:

(1) $$\frac{U_{z}}{U_{ref}}={\left(\frac{z}{z_{ref}}\right)}^{\alpha }$$

while taking a roughness length *α* of 0.1 [35]. The BC for PM_10_ was set to 0 μg m^−3^, since PM_10_ enhancement due to traffic was modeled. Other values for the BC for PM_10_ were considered not appropriate since accurate BC values from measurements are not available. The resultant PM_10_ levels reported here were the CFD-predicted PM_10_ enhancement plus the measured background concentrations. A 24 h simulation was preformed from 9:00 a.m. on 30 November to 8:00 a.m. on 1 December 2009. It was about the middle testing period of the ANN simulation performed above. A model spin-up of 6 h was used prior to the adoption of the CFD model outputs to avoid the influence from model initialization.

Daily traffic was obtained from the annual average data reported by the government’s Transport Department at the roads of concern in 2009 [36]. The model’s default diurnal profile of traffic for an urban road was assumed, with peak hourly daytime traffic flow contributing about 7%. The traffic data at the two major roads [Nathan Road (17,000 vehicles per day) and Lai Chi Kok Road (7000 vehicles per day)] near the AQMS was input into the model. The roads were the only major sources of PM_10_ concentrations measured at the AQMS and were modeled as the line sources. The source height was 0.3 m above the ground. An average emission factor of 105 μg veh^−1^ m^−1^ for PM_10_ [37], which was obtained from observations at different sites, was used.

The model settings are summarized in Table 2.

## 3. Results and Discussion

### 3.1. Results of the ANN Model

**Figure 3:**
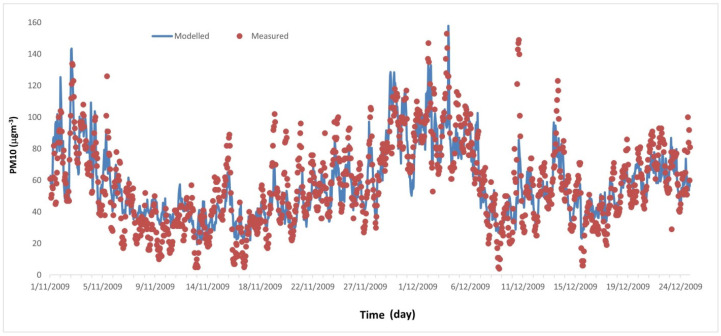
Comparison between the ANN modeling results and the measurements. Temporal variation in PM_10_ as predicted by the ANN model. The measured data are shown as circles.

Figure 3 shows the temporal variation in PM_10_ as predicted by the ANN model during the testing period. The model demonstrates a good performance (r = 0.82, FB = 0.002, RSME = 15.4, MAE = 11.6) and captures the diurnal cycles, the general trend from November to December, and some episodic levels (e.g., on 2 November and 1–4 December).

A series of sensitive tests for the ANN model was performed to determine whether a single input parameter or a combination of them governed the model performance. Prior to the tests, a principal component analysis (PCA) was performed. The PCA results showed that the first four principal components (PCs) accounted for 74% of the total variance (Supplementary Material Table S1). One of the PCs (PC3) showed high loadings (>0.9) with the background PM_10_ and the predicted PM_10_, suggesting a strong association between them. The ANN model construction using only the background PM_10_ as the input parameter could achieve a relatively good model performance (r = 0.77), when compared to the observations. At this point, the result of the PCA was consistent with that of the ANN model’s sensitive test. However, an additional sensitive test by constructing an ANN model using in-canyon wind speed and in-canyon air temperature (essential parameters in the CFD simulation) showed a very poor model performance with r = 0.25. The poor performance might be attributed to the omission of the background PM_10_ levels. Nevertheless, our results suggested that the ML model could perform reasonably well even without the knowledge of traffic data. Such simplification has a major benefit to the practical model application in a smart city, which is discussed in the subsequent sections.

### 3.2. Results of CFD Model

**Figure 4:**
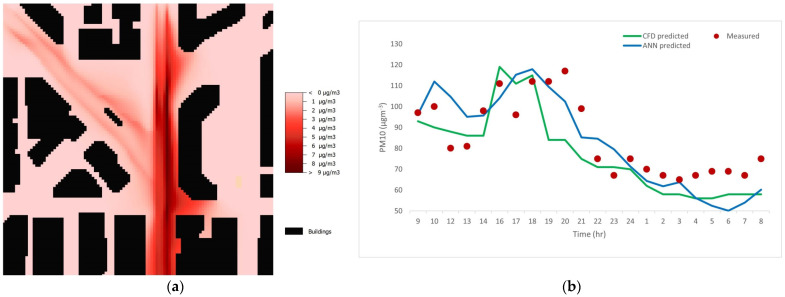
The CFD modeling results and comparison with the ANN modeling results and measurements. (**a**) Sample-predicted distribution of PM_10_ enhancement due to traffic (1.5 m above ground) by the CFD model during peak hours. (**b**) Comparison of the predicted PM_10_ diurnal variations by the CFD model and the ANN model. The measured data are shown as circles.

Figure 4a shows a typical traffic-induced PM_10_ horizontal distribution within the study area as predicted by the CFD model during peak hours. Higher PM_10_ levels near the road sources are clearly depicted under the influence of a weak, northeasterly wind (<0.5 ms^−1^). When compared to the area near Lai Chi Kok Road, the PM_10_ concentrations near Nathan Road are higher because of the higher traffic flow. Specifically, in the morning of 29 November, under the influence of a weak, northerly/northeasterly wind, the monitoring station and nearby areas were at downwind of Nathan Road (Figure 1) and, thus, had relatively high PM_10_ levels (Figure 4b) due to the impact of vehicular pollution plume. At earlier noontime, however, the decreasing PM_10_ levels at the monitoring station and nearby areas were mainly due to the change in wind direction (i.e., southwesterly at noontime) and enhanced vertical mixing with relatively clean air aloft. The CFD results showed that the PM_10_ enhancements due to road traffic during nighttime were very small at most of the areas within the domain (<2 μg m^−3^) because of the low traffic flow. A detailed discussion of the pollution dispersion is not the aim of the current work. Figure 4b shows the diurnal variation in the measured PM_10_ concentrations, as well as the calculated PM_10_ concentrations by the CFD model and ANN model. Daytime-measured PM_10_ concentrations are higher than those at nighttime because of the lower traffic flow at nighttime. The lower measured concentration near noontime is attributed to stronger solar heating that promotes the vertical mixing of pollutants, given a relatively small variation in the daytime traffic flow. In general, the CFD model performs well (Table 3) and captures the temporal variation in the measured PM_10_ levels. Its good performance is likely due to the diurnal profile of traffic assumed in the model, hourly wind speed and direction as the input model boundary conditions, and simulated vertical mixing in the unstable atmosphere near noontime.

The discrepancy in the CFD results for the prediction in the evening hours (1700–1900; Figure 4b) might be attributed to the considerable deviation in traffic flow between the real-time situation and the model’s default profile. Except during 12:00–18:00, the CFD model shows an under-estimation of the measurements most of the time. This under-estimation has been reported elsewhere. Deng et al. [32] pointed out that the under-estimation was profound, especially during days with elevated particulate levels, although the model depicted similar temporal pattern in the measured pollution levels. For a pollution dispersion study from a motorway, De Maerschalck et al. [38] demonstrated a good agreement between the measurements and the modeling results for NO_2_, but not for particulate levels.

While both the ANN and CFD models performed similarly in the PM_10_ predictions studied above (Table 3), the computational time for the ANN model was less than 0.1% of the CFD model. Simulating a one-day hourly PM_10_ variation by the ENVI-met required more than 30 wall-clock hours in parallel processing mode for a computer with four cores, while a ~50-day hourly PM_10_ simulation by the ANN model required less than 30 wall-clock minutes using the same computer. To resolve the demanding computational resource and lengthy time required by CFD simulations, a plausible solution might be a fast-mathematical model with simplified equations for air quality predictions. However, it is well known that a simplified dispersion equation, such as a Gaussian-type equation, performs poorly in dispersion calculations in complicated built environments.

One of the major limitations for the CFD model (and other conventional physiochemical models) in simulating street-canyon air quality is the requirement of real-time traffic counting. Another limitation is that, in reality, it is very difficult to accurately obtain vehicular emission information for all vehicles on the roads in a simulation period. For instance, there are large uncertainties in the vehicular emissions adopted in the model when compared with reality. Actual information, such as emission standards (from EURO-III to EURO-VI), and additional mitigation measures (e.g., diesel particulate filter) fitted at the tailpipe for each vehicle are very difficult (if not impossible) to obtain during routine monitoring. Some studies adopted a vehicular emission model (e.g., Mobile 6 and COPERT4) to better mimic the variation in road traffic emissions and then to feed the information into an air dispersion model, including a CFD model [39,40,41]. However, this kind of model requires many inputs, such as fuel consumption, fleet configuration, trip length, distribution of vehicle miles traveled by road types, average speed distribution by road types, annual mileage, which are not available in many areas/countries; thus, large uncertainty in the simulated traffic emissions and, in turn, the air quality simulations results. This poses a challenge in using a vehicular emission model to obtain relatively accurate results for practical use in an urban environment.

Besides that, the good performance of the CFD model is likely due to the diurnal profile of traffic adopted and the use of hourly wind speed and direction as the model boundary conditions. On the contrary, many research efforts available in the literature, for the purpose of scenario simplification, adopted constant emissions and boundary conditions (e.g., for wind), without considering unstable atmospheric conditions. It demonstrates that the practical use of a CFD modeling technique as an air quality management tool for the urban neighborhood-scale air pollution problem is, in general, very difficult.

## 4. Conclusions

In this study, the ANN approach, as an ML algorithm, was used to make PM_10_ predictions near road traffic emissions in the UCL. The performance of the ANN model was further compared with the CFD model. Both the ANN and CFD models performed similarly when their predictions were compared with the measurements. However, the ANN model is much faster and requires less computational resources and fewer input parameters. The last factor might be critical in the context of air quality management for a smart city. For instance, acquisition of accurate real-time vehicle emission factors is difficult for CFD simulations, but traffic flow and emission factors are not required for the ANN model simulations based on the finding of the current study. These issues have been discussed in more details. Nevertheless, one of the strengths of the CFD model is that it provides the spatial dynamics of urban air pollution, which is difficult to obtain with the currently formulated ANN model.

The ANN model adopted in our study demonstrates its usefulness in air quality predictions, especially as a useful tool for smart city applications. It provides acceptable results in both neutral and unstable atmospheric conditions, whereas additional complicated model settings/assumptions are required for the CFD model to simulate the conditions in an urban environment. Nevertheless, the ANN model, like other ML models, is a so-called “black-box”, which has limited contribution to knowledge development of physical processes and interaction of the driving mechanisms related to dispersion within urban street canyons. This issue may need further research in future.

## Figures and Tables

**Table 1:** The ANN model settings.

	Parameters
Input layer	Number of neurons: 11 Background wind speed Background wind direction Background air temperature Background PM_10_ concentration Atmospheric pressure Rainfall Canyon wind speed Canyon wind direction Canyon air temperature Dates of a week Weekday/weekend
Hidden Layer	Number of neurons: N_hidden_ = 2N_input_ + 1
Output Layer	Number of neurons: 1
Transfer function for hidden layer	Tangent Sigmoid
Transfer function for output layer	Linear
Training method	Goal: minimum MSE Epoch: 1000 times Algorithm: Levenberg–Marquardt
Dataset	Total size: 8616
	Data for training: 70%
	Data for validation: 15%
	Data for testing: 15%

**Table 2:** The CFD model settings.

Parameters	Remarks/Values ^1^
Meteorological conditions (wind speed, wind direction, relative humidity, and air temperature)	Hourly local measurements
Boundary condition for PM_10_ Pollution source Source emission factor for PM_10_	0 μg m^−3^, since PM_10_ enhancement due to traffic was modeled Line sources with a height of 0.3 m above the ground 105 μg veh^−1^ m^−1^

^1^ See text for details.

**Table 3:** Summary of model performance.

Model	R	FB	RMSE	MAE
ANN	0.84	0.02	12.2	10.4
CFD	0.81	0.09	13.7	11.3

## Data Availability

Please refer supplementary data of the study in Supplementary Material.

## Supplementary Materials

The following supporting information can be downloaded at https://www.mdpi.com/article/10.3390/ijerph20032412/s1, Table S1: Principal component analysis for the association between input parameters.
