# Supplementary material for: Application of a Machine Learning Method for Prediction of Urban Neighborhood-Scale Air Pollution
Source: Int J Environ Res Public Health. 2023 Jan 29;20(3):2412. doi: 10.3390/ijerph20032412 (PMC9915966; doi:10.3390/ijerph20032412)
Supplement: Supplementary file 1 [file ijerph-20-02412-s001.zip › ijerph-2126307-supplementary.pdf]

Table S1. Principal component analysis for the association between input parameters.

	Component 1	Component 2	Component 3	Component 4
Canyon air temperature	**0.98**	**-0.68**	0.01	0.01
Background air temperature	**0.98**	-0.04	0.01	-0.01
Atmospheric pressure	**-0.86**	-0.03	0.15	-0.08
Background wind speed	-0.17	**-0.80**	-0.05	-0.08
Canyon wind speed	0.19	**0.61**	0.22	-0.30
Rainfall	-0.01	**0.52**	-0.10	0.36
Canyon PM_{10}	0.02	-0.02	**0.97**	0.08
Background PM_{10}	-0.13	0.02	**0.92**	0.04
Canyon wind direction	-0.06	-0.25	0.07	**0.76**
Background wind direction	0.15	0.12	0.03	**0.76**

Absolute value > 0.5 is bolded.
